# Cross-situation consistency of mobile App users’ psychological needs

**DOI:** 10.1371/journal.pone.0215819

**Published:** 2019-04-24

**Authors:** Zaoyi Sun, Pei Zhang, Zhiwei Ji, Chuansheng Chen, Qun Wan, Xiuying Qian

**Affiliations:** 1 Department of psychology and Behavioral Sciences, Zhejiang University, Hangzhou, Zhejiang Province, China; 2 College of Information Science & Electronic Engineering, Zhejiang University, Hangzhou, Zhejiang Province, China; 3 Department of Psychological Science, University of California Irvine, Irvine, California, United States of America; 4 Zhejiang Big Data Exchange Center, Tongxiang, Jiaxing, Zhejiang Province, China; Western Sydney University, AUSTRALIA

## Abstract

Previous studies showed that individuals’ traits could be used to explain the similarity of behavioral patterns across different occasions. Such studies have typically focused on personality traits, and have not been extended to psychological needs. Our study used a large dataset of 1,715,078 anonymous users’ App usage records to examine whether the individual’s needs-based profiles of App usage were consistent across different situations (as indexed by categories of App functions). Results showed a high level of consistency across situations in a user’s choice of Apps based on the needs the Apps could satisfy. These results provide clear evidence in support of cross-category App recommendation systems.

## Introduction

Humans have a set of universal basic needs that play important functions in daily life [1]. As one of the earliest theorists of human needs, Henry Murray in 1938 identified five categories of 17 needs: ambition, materialism, power, affection, and information [1]. In 1954, Abraham Maslow conceptualized five levels of human needs: physiological, safety, social (love and belonging), esteem, and self-actualization needs [2]. In 1967, David McClelland identified three needs (achievement, affiliation, and power) as the main motivators for human behavior [3]. Recently, Edward Deci and Richard Ryan proposed self-determination theory that focuses the needs for competence), autonomy, and psychological relatedness [4, 5]. However the structure of needs is conceptualized, there is a general consensus among researchers that psychological needs are deep-rooted in our evolutionary history but serve as the driving force behind modern human behaviors [6, 7].

Although human needs are universal, stable biological factors such as genes and sex and relatively stable individual and socio-cultural factors such as current life stage, life history strategy, and culture help shape the strength and workings of various needs [8, 9]. According to Henry Murray, such individual differences in the importance of various needs help to shape individuals’ unique personality [1]. Murray’s view has recently been summarized succinctly as “our personalities are a reflection of behaviors controlled by needs” [10]. Surprisingly, however, individual differences in needs have rarely been studied in terms of their stability or trait-likeness, in clear contrast with previous research focusing on personality traits [11–14].

Thus far only a few domain-specific needs have been examined for their stability over time and across situations (i.e., different social settings, physical environments, or performing different tasks). One domain is school achievement. Specifically, one previous study of the need for achievement (or achievement motivation) found that the orientation of intrinsic vs. extrinsic motivation appeared to be trait-like because students consistently reported the same motivation orientation across four different academic subjects [15]. Another study reported that the need for achievement was highly related to the trait of self-efficacy (i.e., the tendency to view oneself as capable of meeting task demands in a wide variety of situations) [16]. Extending achievement motivation in the school setting to that in the work place, Kanfer and Heggestad specifically treated work motivation as a trait that can be used for personnel selection [17]. To our knowledge, however, no study has examined the stability of broader categories of needs such as those articulated by Maslow.

The current study aimed to examine the cross-situation consistency/stability of Maslow’s psychological needs associated with mobile App usage. Specifically, we used a large dataset of App usage records to examine whether the needs-based profiles of App usage were consistent across different situations (as indexed by categories of App functions, to be explained below). The use of App usage data to study psychological needs represents a novel approach enabled by recent advances in big data science and has two clear advantages over traditional psychological studies using questionnaires in terms of sample sizes and source of data (actual behaviors rather than self-reports). Similar studies have used data of digitally mediated behaviors to successfully classify individuals by their characteristics such as gender, age, and personality traits [18, 19, 20].

The current study is an extension of a previous study that developed a method to automatically label mobile Apps in terms of whether and to what extent they can satisfy users’ particular psychological needs [21]. In that study, the researchers obtained in-depth interview data from App users about what needs were satisfied by the Apps they used and crawled the App stores for user reviews for similar information. Using grounded theory and the related procedures of substantive and theoretical coding of the data, the researchers identified eight types of psychological needs related to App use: utilitarian (e.g., increasing work efficiency and saving time), low-cost (e.g., inexpensive), security (e.g., ensuring information security, privacy), health (e.g., tracking the physical state and health-related data), hedonic (e.g., fun), social (e.g., facilitating communication with others), cognitive (e.g., satisfying curiosity), and self-actualization (e.g., improving himself/herself) needs. Through text analysis of user reviews of the Apps and with the use of machine learning algorithms, each review was automatically provided with multiple labels of the types of needs the App was able to satisfy. In the current study, we combined three sources of data (the type of needs each App satisfies, the users’ actual App usage records, and the broad categories of Apps as defined by the App stores) to examine cross-situation consistency of users’ needs. It should be noted that in this study we only focused on cross-situation consistency of psychological needs related to App use, not cross-time stability because our data did not have the time span needed to examine temporal stability of traits (months or years).

To index the extent to which psychological needs were being satisfied, we used the number of times each App was used. We assumed that the greater an individual’s particular need was, the more times he/she would use an App that was able to satisfy that need [22]. To index situations, we relied on the large categories of Apps’ functions with the assumption that different situations typically require the use of different types of Apps. For example, users are likely to use mapping and navigation Apps when traveling; shopping-related Apps when shopping; restaurant-related Apps when eating out, etc. Finally, to determine cross-situation consistency of psychological needs, we relied on the fact that (1) Apps of the same large category (e.g., navigation, news) can meet different types of psychological needs to different extents (e.g., utilitarian, cost, social) and (2) individuals use multiple Apps within and across situations (or large categories of Apps). To illustrate, a person with stably higher social needs is more likely to choose navigation Apps with relatively higher social needs scores and to choose news service Apps with relatively higher social needs scores. Based on the above discussion about the trait-likeness of human needs, we expected to find evidence for cross-situation consistency of psychological needs related to App usage.

## Materials and methods

### Data sources

This research was approved by the Human Research Ethics Committee of the Department of Psychology and Behavioral Sciences, Zhejiang University. The need for participant consent was waived by the committee as we adopted millions of users’ anonymous data, and presented only aggregated results. The following three sources of data were used: (1) The original user data provided by a telecommunications operator was used to compile the number of times each user used each App, (2) The 12 App function category tags from Huawei App store were used to index situations, and (3) Each App’s needs tags from a previous study [21] were used to index the needs it could satisfy.

### The original user data (user behavior)

When users use mobile phones, the communications operator logs three types of data: call records (Voice), Short Message Service records (SMS), and Internet visiting records (Data). Our study used only the Data, from which we extracted information about the internet sites accessed by the users. Note that the Data did not contain users’ specific communication content and interactive data. The operator stores log data for a period of time for the purposes of charging its customers or network maintenance. We used the 20-day log data from July 9, 2016, to July 28, 2016, provided by a telecommunications operator in Shanghai, China. The total number of users of our data was 2,917,800. We adopted the following measures to protect users’ privacy [23]. (1) Anonymization: All the telephone numbers involved were encrypted into unique strings, so the data could not be matched with specific users; and any other fields that might display user identity were removed. (2) Minimal information principle: Internet visiting records did not contain specific communication content, interactive content, and other private data, as mentioned earlier, and furthermore the location of the record was the base station, not the actual GPS coordinates of the user at a specific time. Moreover, no demographic information about the users was available. (3) Aggregated results: We presented only aggregated results, not those of individual users. For the current study, the only user behavior data we used were the number of times each App was used by each user.

### App function category tags (situations)

App function category tags were based on the Huawei App market for 12 App categories, including news service, navigation, shopping and so on. As Huawei is one of the top two smartphone makers in China, Huawei App market has a large number of users. We collected 15000 Apps with their corresponding function category tags: each App *k*(0≤*k*≤*K*,*K* = 15000) had a function category tag $f^{k}\in \mathbb{Z}$, 0≤*f*^*k*^≤11.

#### App needs tags

As mentioned earlier in the Introduction section, using semi-structured in-depth interviews and App reviews, we conducted substantive and theoretical coding of the data and identified eight types of psychological needs commonly expressed by App users: utilitarian, low-cost, security, health, hedonic, social, cognitive, and self-actualization needs [21]. Words and phrases that indicated the different types of needs formed a corpus used to build training samples. Then latent Dirichlet allocation (LDA) and support vector machine (SVM) algorithms were used to filter reviews in terms of whether they included needs-related comments. Finally, Labeled-LDA was used to automatically provide each review with multiple labels of the types of needs mentioned and based on the reviews, the Apps were then labeled by the different types of needs they satisfied.

In the current study, each App *k*(0≤*k*≤*K*,*K* = 15000) had a needs score vector $E^{k}={\left[e_{0}^{k},e_{1}^{k},\ldots ,e_{7}^{k}\right]}^{T}$, where $0\leq e_{i}^{k}\leq 1$, $\sum_{i=0}^{7}e_{i}^{k}=1$.

### Methods

**Fig 1:**
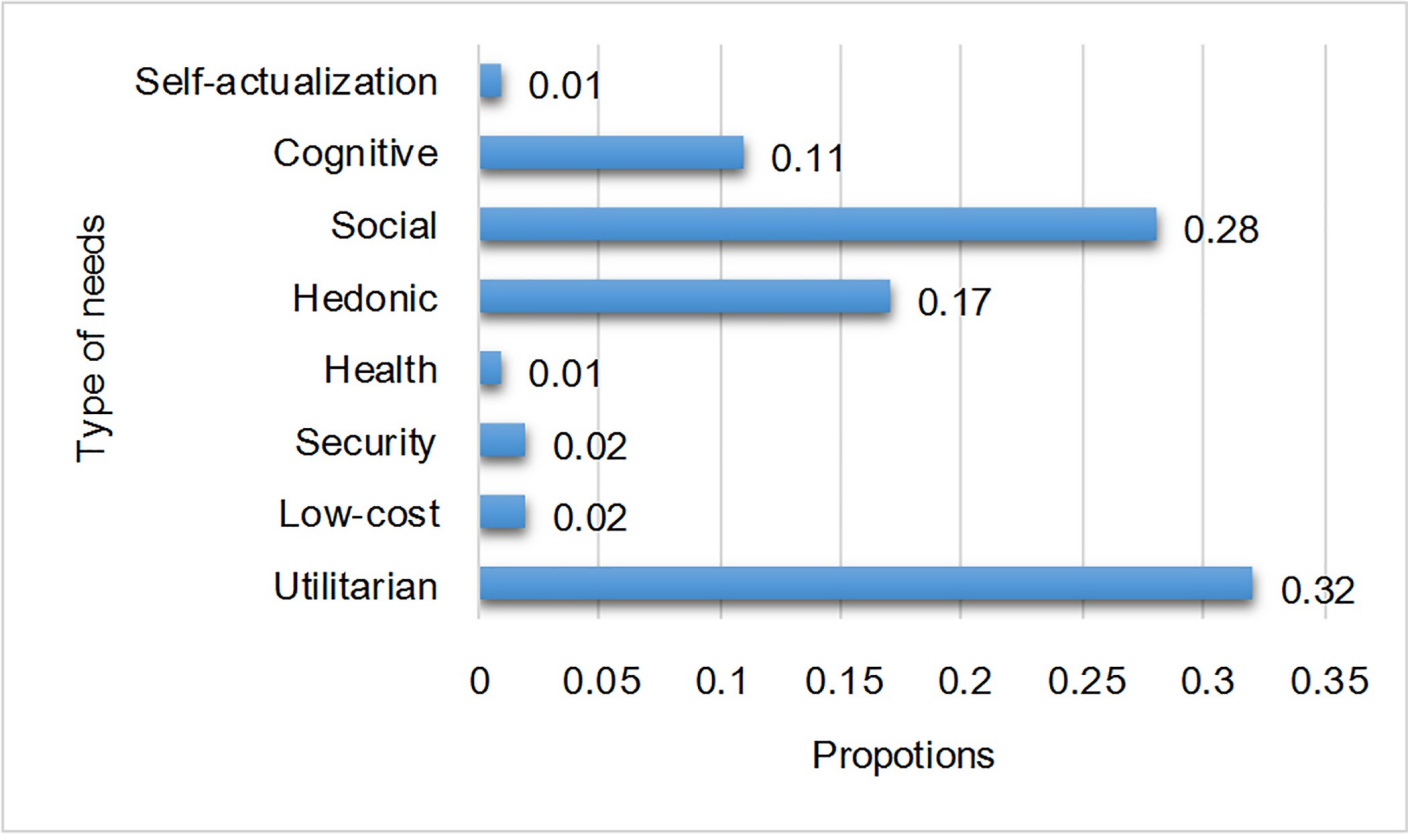
The procedure used to examine cross-situation consistency of App users’ needs.

After obtaining the above data, we followed the steps below to evaluate users’ needs profiles and their consistency across situations (see Fig 1).

1. **Data integration.** The original data provided by communications operator included all the host sites visited by the users. With an App-Host matching library, we were able to match 2,917,800 App usage records with host sites. Since the purpose of our study was to evaluate the consistency of users’ needs across different categories of App functions, the users who had usage records for few (< 4) App function categories (or situations) were deemed as not having diverse enough situations, so these users’ data were excluded from further analysis. The resulting number of users with analyzable data was 1,715,078 and denoted by N. We aggregated each user *n*(0≤*n*≤*N*) and each App k’s usage data and obtained the App usage matrix $C\in {\mathbb{N}}^{N\times K}$, *C* = {*C*_*nk*_|*C*_*nk*_≥0}, where *C*∈*N*^N*K^, *C* = {*C*_*nk*_|*C*_*nk*_≥0}.
  The data format after convergence was:
  User_id | App_id | Function_tag | Needs_tag | Use_times (*n*|*k*|*f*^*k*^|*E*^*k*^|*C*_*nk*_)
2. **Evaluation of all users’ needs.** Following the same procedure used in a previous study’s [21] discussed in the Introduction, we calculated each needs type’s (dimension’s) weighted mean score T (weighted by each App’s total usage), where:
  $$T_{i}=\frac{\sum_{n=o}^{N‐1}\sum_{k=0}^{K-1}C_{nk}E_{i}^{k}}{\sum_{n=0}^{N-1}\sum_{k=0}^{K-1}C_{nk}}(i=0,1,\ldots ,7)$$
3. **Individual users’ needs vector for each situation (i.e., App function category).** Weighted by individual users’ usage of each App, we calculated individual users’ weighted mean score of each needs dimension in a specific App function category. For each user n, his /her needs vector *S*_*np*_ in App function category *p*(0≤*p*≤11) was:
  $S_{npi}=\frac{\sum_{k=0}^{K-1}C_{nk}E_{i}^{k}\delta (f^{k}-p)}{\sum_{k=0}^{K-1}C_{nk}\delta (f^{k}-p)},(i=0,1,\ldots 7),f^{k}\in \mathbb{Z}$, where $\delta (x)=\left\{ \begin{array}{l} 1,x\equiv 0\\ 0,otherwise \end{array}\right.$
4. **Evaluation of each App function category’s needs.** For each App *k*(0≤*k*≤*K*), the total usage time was calculated by $C_{k}=\sum_{n=0}^{N-1}C_{nk}$, (0≤*k*≤*K*). Then weighted by each App’s usage, each needs dimension’s weighted mean score vector *μ*_*p*_ of a given App function category was calculated as $\mu_{pi}=\frac{\sum_{k=0}^{K-1}C_{k}E_{i}^{k}\delta (f^{k}-p)}{\sum_{k=0}^{K-1}C_{k}\delta (f^{k}-p)}$, and the corresponding standard deviation vector *σ*_*p*_ was calculated as:
  $$\sigma_{pi}=\sqrt{\frac{\sum_{k=0}^{K-1}C_{k}{\left(E_{i}^{k}-\mu_{pi}\right)}^{2}\delta (f^{k}-p)}{\sum_{k=0}^{K-1}C_{k}\delta (f^{k}-p)}},\;(i=0,1,\ldots ,7).$$
5. **Individual users’ needs z-score vector of each App function category.** Because the mean needs dimensions are likely to differ by situation/category, users’ needs scores needed to be standardized within each situation/category. For each user n, we calculated the needs z-score vector *z*_*np*_ in App function category p as: $z_{npi}=\frac{S_{npi}-\mu_{pi}}{\sigma_{pi}}$, (*i =* 0,1,…,7). The needs z-score vector for a specific App function category reflected the relative (standardized) distance of each needs dimension between the user’s needs level and the function category’s average needs level. This method underscored the user’s differentiated needs in an App function category.
6. **Evaluation of cross-situation consistency of users’ needs.** Finally, to assess the cross-situation/category consistency of users’ needs, we relied on the calculation of Euclidean space and distance. For each user *n*, each of his/her used App function category had a corresponding *z*_*np*_, which formed a Euclidean space. We calculated the center of the space as *center*_*n*_:
  $$center_{ni}=\frac{\sum_{p=0}^{11}Z_{np}}{12-\sum_{p=0}^{11}\delta (Z_{np})}.$$

Then we calculated the *d*_*np*,_ which represented the distance from the user’s *z*_*np*_ of each App function category *p*(0≤*p*≤11) to the *center*_*n*_: *d*_*np*_ = ‖*Z*_*np*_−*center*_*n*_‖_2_. Finally we averaged these distances of a user by: $d_{n}=\frac{\sum_{p=0}^{11}d_{np}(1-\delta (Z_{np}))}{12-\sum_{p=0}^{11}\delta (Z_{np})}$, and this index measured a user’s deviation from the needs *center*_*n*_.

## Results

### The overall distribution of user needs

**Fig 2:**
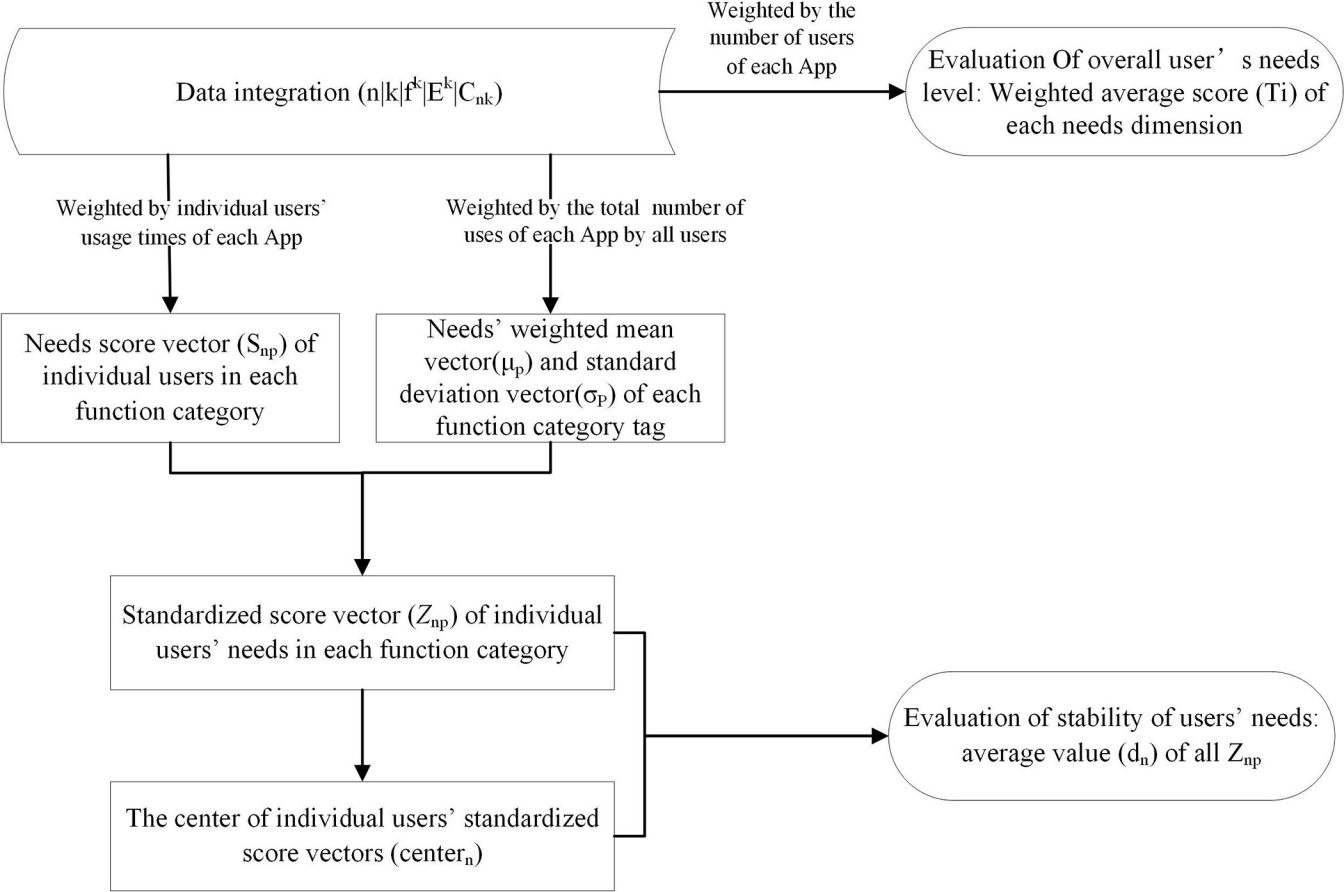
Distribution of the eight types of needs.

Fig 2 shows the overall distribution of user needs. The proportion of utilitarian needs was the highest, followed by that of social needs, which was higher than the proportions of hedonic and cognitive needs. The other needs were low in frequency including low-cost, health, security, and self-actualization. These results were similar to those of previous work in terms of the high proportions of utilitarian needs (39% in current study vs. 44% in [21].) and low proportions of security, health, and self-actualization (less than 2% in both current study and previous study [21]), as well as somewhat low proportions of low-cost needs (2% vs. 7%) and cognitive needs (11% vs. 6%). The main differences between the two studies were in hedonic needs (17% vs. 30%) and social needs (28% vs. 10%). These differences can be explained by the use of two different indices for psychological needs: The current study used the actual number of times the Apps were used whereas the previous study [21] used the number of times the Apps were downloaded. It appears that Apps that can satisfy hedonic were more likely to be downloaded but less likely to be used, whereas Apps that can satisfy social needs were more likely to be used even though they had fewer downloads.

### Cross-situation consistency of individual users’ needs

**Fig 3:**
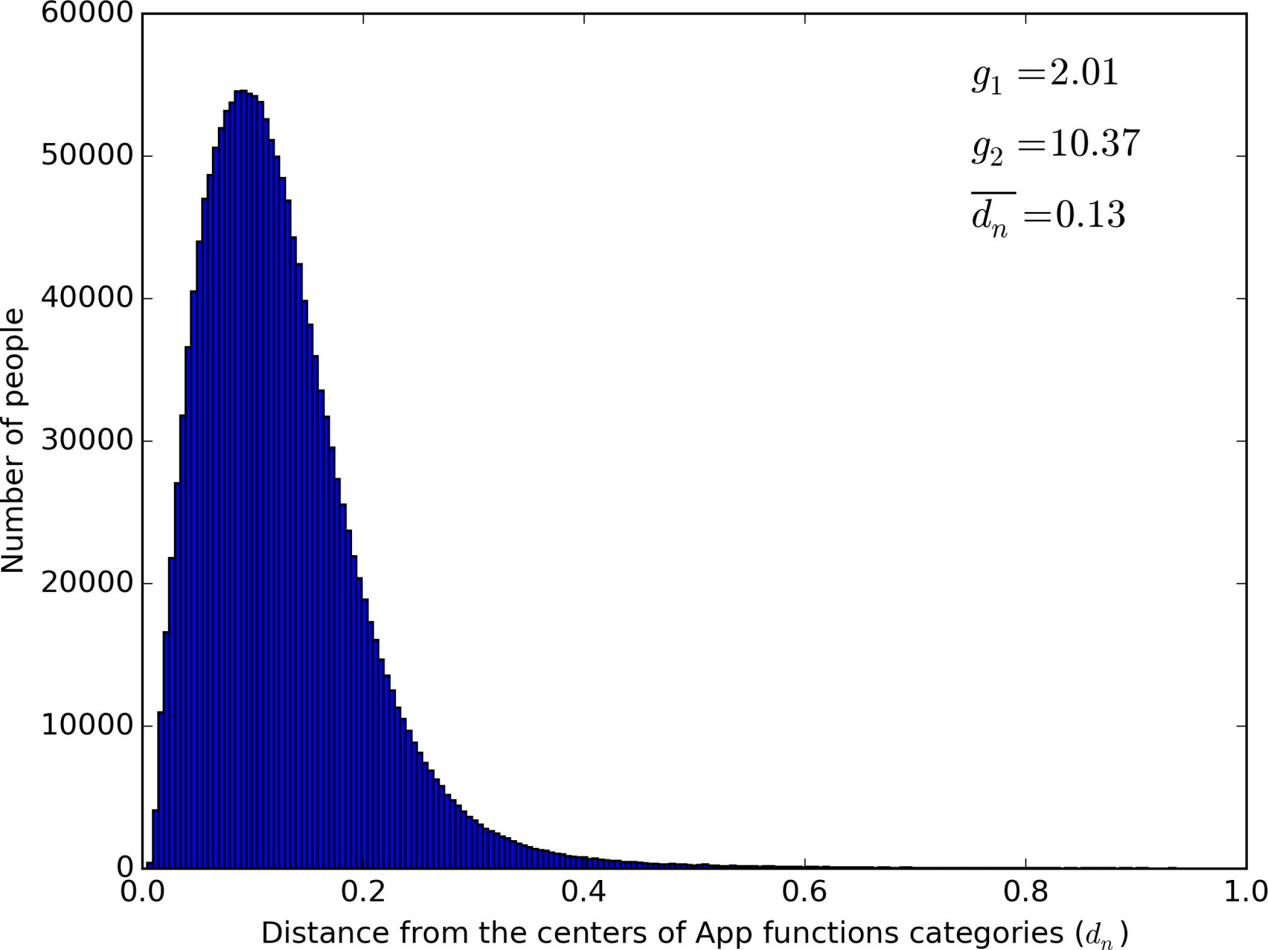
Overall distribution of individual users’ distance from the centers of App functions categories (*d*_*n*_)(where *g*_1_: Coefficient of skewness, *g*_2_: Coefficient of kurtosis, $\bar{d_{n}}$: The average value of *d*_*n*_).

Following the steps 1–5 described above, we obtained the distribution of *d*_*n*_, which represented the average distance of individual users’ needs score vector from the centers of those vectors. The overall distribution of *d*_*n*_ is shown in Fig 3. We used Python 3 to calculate the skewness (*g*_1_), kurtosis (*g*_2_), mean $\left(\bar{d_{n}}\right)$, and standard deviation of *d*_*n*_. Results showed that the distribution of *d*_*n*_ was somewhat left-skewed (*g*_1_) and had high kurtosis (*g*_2_), with a mean of 0.13, *SD* = 0.08, suggesting that most users’ average distances from the centers of App function categories were small (i.e., high consistency of needs across App function categories).

**Fig 4:**
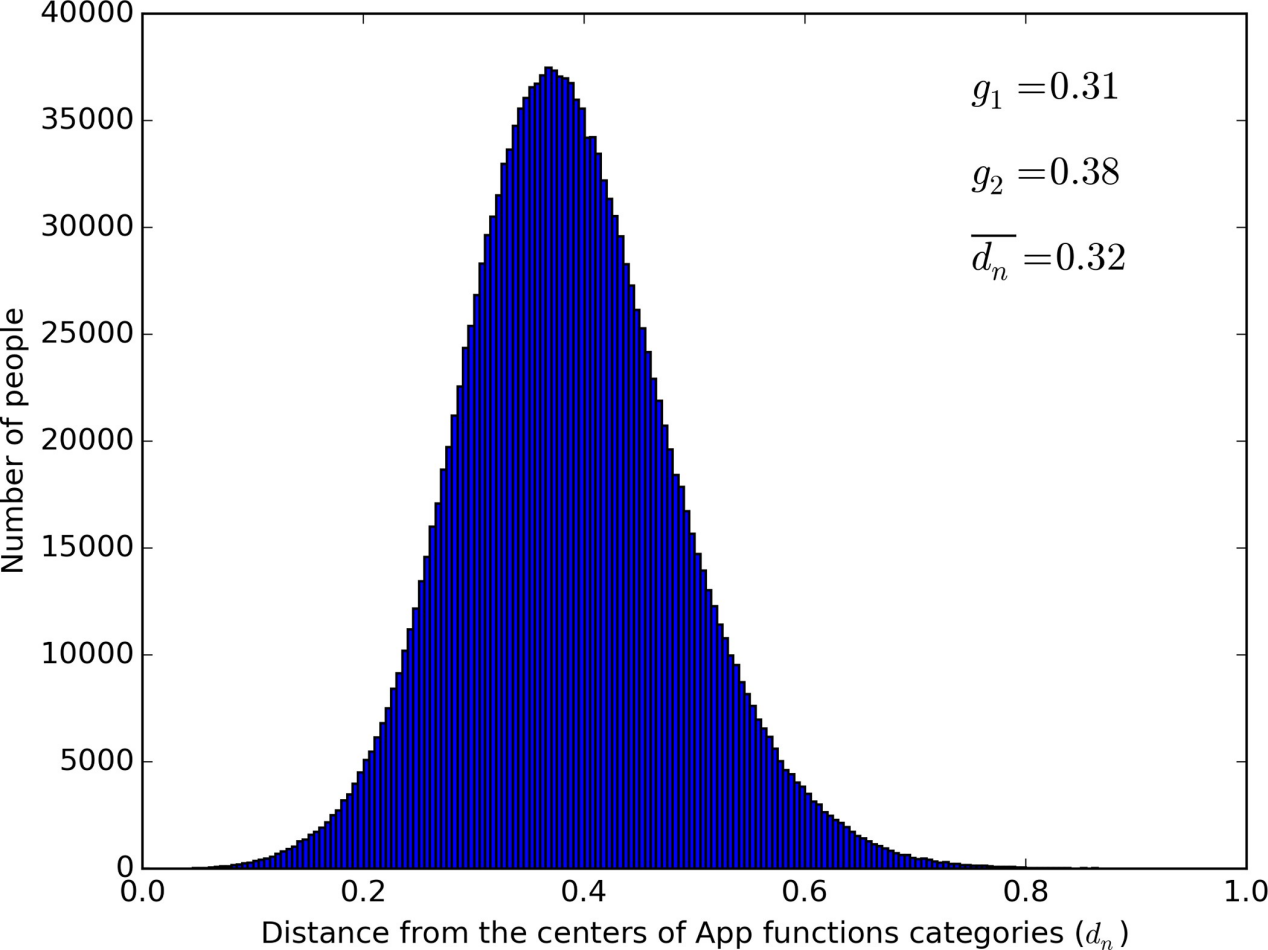
The results of the randomized sampling procedures (where *g*_1_: Coefficient of skewness. *g*_2_: Coefficient of kurtosis, $\bar{d_{n}}$: The average value of *d*_*n*_).

To further examine the level of consistency based on the above analysis, we established a baseline distribution based on randomized data. For each App function category with usage records, m Apps (the number of Apps the user actually used in the given function category) were randomly selected from the category’s total number of Apps. The usage (number of times) of these Apps was randomly matched with the selected m Apps to generate a baseline distribution following the same analytical procedure as used above. As Fig 4 shows, the distribution of the randomized data was normal with a mean distance of 0.32, SD = 0.10, and low skewness and kurtosis.

To compare the two distributions, we conducted two sets of analyses. First, we used Chi square goodness of fit test by binning (or grouping) the continuous data of distance into discrete groups with the following steps:

1. Determination of the number of bins (groups) and each bin’s range. In general, 10–15 bins are sufficient to preserve the data’s distribution information, but because of the large amount of data and the great spread of the two distributions, we used 50 bins.
2. Calculation of each bin’s frequencies (number of users for a given bin): *f*_0_ for the actual data and *f*_e_ for the randomized data. Because the bins at the two ends had low frequencies (< 5 users), they were combined into the neighboring bins, yielding a final number of 46 bins.
3. Determination of the degrees of freedom. The degrees of freedom (df) were 46–1 = 45.
4. Calculation of Chi square. Pairs of *f*_0_, *f*_*e*_ were entered into the formula: $\chi^{2}=\sum \frac{{\left(f_{0}-f_{e}\right)}^{2}}{f_{e}}$ yielding *χ*^2^ = 1.31×10^9^, $\chi_{0.005}^{2}(df=50)=79.5$.
5. Calculation of the effect size. The effect size (*d*) was calculated as $d=\sqrt{\frac{\chi^{2}}{N}}$ where N was the total number of users (N = 1,715,078). We calculated d = 28.6, suggesting an extremely large effect (as Cohen's' *d*> 0.8 is large).

Second, we used *Z*′ significance test to evaluate the mean difference between the two distributions (one of which was not normal). We calculated *Z*′ = 2254.5 (*p* < .001). The effect size (*d*') was 2.39, also suggesting a large effect (as Cohen's' *d*> 0.8 is large).

## Discussion

Using three sources of data, namely, App’s needs probability distribution from [21], anonymous users’ App usage records, and the functional categories of the Apps, we found that most users showed a high level of consistency in their needs profiles (or the traits of psychological needs) across different situations (indexed by App functional categories). The results demonstrated that App users’ needs profiles may be considered as stable traits, which can be used to predict user behaviors and to develop App recommendation systems across categories.

Understanding how persons and situations contribute to behavioral consistency is a central goal for the science of behavior [14]. There have been fierce debates between the “person” (personality psychology) and the “situation” (social psychology) perspectives. The “person” argument claims that individual differences are stable and trait-like, which can be used to predict behaviors across time and situations [13]. The “situation” argument claims that situation drives behavior so individuals’ behavior is highly variable across situations and time. Our results provided evidence for the “person” perspective in terms of psychological needs being satisfied by App usage across situations.

Cross-situation consistency of App usage may have both biological (such as temperaments) and social cognitive causes, just as is the case for other traits such as personality. Researches have proposed the classic psychobiological model of temperaments and characters, which has been widely adopted in genetic research of personality traits [24]. Similarly, from the perspective of evolutionary biology, individuals’ needs systems were evolved to deal with different functional threats and opportunities. These systems also interact with situational inputs in functionally adaptive ways [25], which, as emphasized by social-cognitive theory, would necessarily involve cognitive processes such as expectations, values, goals, and self-management strategies. In sum, previous research has focused on personality as stable traits [11–14, 24], but our research extended this line of research to needs traits in App users using a big data approach.

The current study relied on the massive App usage data across situations. Consequently, results of such research should not only help us to understand human behavior in general but also has specific practical implications. For example, such information can be used to improve products and services. By adding psychological needs dimensions to the current user profile models, recommendation systems can improve their precision, especially for cross-category recommendation systems. For example, a user who has a high level of social needs can be given recommendations of Apps that are high in satisfying social needs regardless of the Apps’ main functional category. This type of psychological advertising has been discussed by Wang et al. [26], who defined five levels of needs, each represented as a set of textual patterns, and applied them to the learning framework of ad-click prediction. The results showed that needs features could significantly increase the accuracy of ad-click prediction.

Although our approach provided evidence of the cross-situation consistency or trait-likeness of App users’ needs, several limitations of the current study need to be discussed. First, we only examined cross-situation consistency of users’ needs profiles with 20-day log data. Therefore, we could not evaluate cross-time stability of users’ needs profiles. Second, in the research using big data, the protection of user privacy has increasingly become a concern [27]. Some researchers stated that when users realized that the service providers can make precise predictions of their personal traits, they may distrust or reject digital technologies [19]. Although this study only used anonymous users’ App usage data, the service provider would have access to more comprehensive usage data, and hence users’ privacy concern may become a bigger issue when the service provider begins to use sophisticated methods to profile their users. A balance has to be maintained between the improvement of customer service (and the understanding of human behavior in general) and the concern for privacy. Finally, our study focused on the stability of needs without considering other traits such as personality. Although needs are the driving force behind personality and are hence more basic than the latter as discussed earlier, future research should examine relative stabilities of needs related to App usage and personality traits (if the content of the information accessed or provided by the users would allow us to determine personality traits reliably).

## References

[1] Murray HA. Explorations in Personality: A Clinical and Experimental Study of Fifty Men of College Age. American Sociological Review. 1938 10.2307/2084329

[2] Maslow AH. Motivation and Personality. Motiv Personal. 1954; 62–27. 10.1037/h0039764

[3] Mcclelland D. C. (1967). THE ACHIEVING SOCIETY. The achieving society /.

[4] Ryan RM, Deci EL. Self-determination theory and the facilitation of intrinsic motivation, social development, and well-being. Am Psychol. 2000;55: 68–78. 10.1037/0003-066X.55.1.68 11392867

[5] Baard PP, Deci EL, Ryan RM. Intrinsic Need Satisfaction: A Motivational Basis of Performance and Well-Being in Two Work Settings. J Appl Soc Psychol. 2004;34: 2045–2068. 10.1111/j.1559-1816.2004.tb02690.x

[6] Baumeister RF, Leary MR. The need to belong: desire for interpersonal attachments as a fundamental human motivation. Psychol Bull. 1995;117: 497–529. 10.1037/0033-2909.117.3.497 7777651

[7] Sheldon KM, Elliot AJ, Kim Y, Kasser T. What Is Satisfying About Satisfying Events ? Testing 10 Candidate Psychological Needs. J Pers Soc Psychol. 2001;80: 325–339. 10.1037//O022-3514.80.2.325 11220449

[8] Mahmoud O. The rational animal: How evolution made us smarter than we think. Int J Mark Res. 2016;58: 333–335.

[9] Griskevicius V, Kenrick DT. Fundamental motives: How evolutionary needs influence consumer behavior. J Consum Psychol. 2013;23: 372–386. 10.1016/j.jcps.2013.03.003

[10] Cherry K. Murray's Theory of Psychogenic Needs. Personality psychology. 2018 Available from http://psychology.about.com/od/theoriesofpersonality/a/psychogenic.htm.

[11] Shoda Y, Mischel W, Wright JC. Intraindividual stability in the organization and patterning of behavior: incorporating psychological situations into the idiographic analysis of personality. [Internet]. Journal of personality and social psychology. 1994 pp. 674–87. 10.1037/0022-3514.67.4.674 7965613

[12] Fleeson W. Towards a structure and process integrated view of personality: Traits as density distributions of state [Internet]. Journal of Personality and Social Psychology. 2001 pp. 1011–1027. 10.1037/0022-3514.80.6.1011 11414368

[13] Fleeson W. Moving personality beyond the person-situation-debate. Curr Dir Psychol Sci. 2004;13: 83–87.

[14] Leikas S, Lönnqvist J, Verkasalo M. Persons, Situations, and Behaviors: Consistency and Variability of Different Behaviors in Four Interpersonal Situations. J Pers Soc Psychol. 2012;103: 1007–1022. 10.1037/a0030385 23066881

[15] Harter S, Jackson BK. Trait vs. nontrait conceptualizations of intrinsic/extrinsic motivational orientation. Motiv Emot. 1992;16: 209–230. 10.1007/BF00991652

[16] Chen G., Gully S. M., Whiteman J. A., & Kilcullen R. N. (2000). Examination of relationships among trait-like individual differences, state-like individual differences, and learning performance. Journal of Applied Psychology, 85(6), 835 11125649 10.1037/0021-9010.85.6.835

[17] Kanfer R, Heggestad ED. Motivational traits and skills: A person-centered approach to work motivation. Res Organ Behav Vol 19, 1997. 1997;19: 1–56. 10.1007/s13398-014-0173-7.2

[18] Kosinski M, Stillwell D, Graepel T. Private traits and attributes are predictable from digital records of human behavior. Proc Natl Acad Sci U S A. 2013;110: 5802–5. 10.1073/pnas.1218772110 23479631 PMC3625324

[19] Youyou W, Kosinski M, Stillwell D. Computer-based personality judgments are more accurate than those made by humans. Proc Natl Acad Sci. 2015;112: 1036–1040. 10.1073/pnas.1418680112 25583507 PMC4313801

[20] Thorstad R, Wolff P. A big data analysis of the relationship between future thinking and decision-making. Proc Natl Acad Sci. 2018;115: E1740–E1748. 10.1073/pnas.1706589115 29432182 PMC5828570

[21] Sun Z, Ji Z, Zhang P, Chen C, Qian X, Du X, et al Automatic labeling of mobile apps by the type of psychological needs they satisfy. Telemat Informatics. 2017;34: 767–778. 10.1016/j.tele.2017.03.001

[22] Chen GM. Tweet this: A uses and gratifications perspective on how active Twitter use gratifies a need to connect with others. Computers in Human Behavior. 2011 pp. 755–762. 10.1016/j.chb.2010.10.023

[23] Becker R a., Cáceres R, Hanson K, Isaacman S, Loh JM, Martonosi M, et al Human mobility characterization from cellular network data. Commun ACM. 2013;56: 74 10.1145/2398356.2398375

[24] Cloninger CR, Svrakic DM, Przybeck TR. A psychobiological model of temperament and character. Arch Gen Psychiatry. 1993;50: 975–90. 10.1001/archpsyc.1993.01820240059008 8250684

[25] Sherman RA, Nave CS, Funder DC. Situational similarity and personality predict behavioral consistency. J Pers Soc Psychol. 2010;99: 330–343. 10.1037/a0019796 20658847

[26] Wang T, Bian J, Liu S, Zhang Y, Liu T-Y. Psychological advertising: Exploring User Psychology for Click Prediction in Sponsored Search. Proc 19th ACM SIGKDD Int Conf Knowl Discov data Min—KDD ‘13. 2013; 563. 10.1145/2487575.2487699

[27] Conger S, Pratt JH, Loch K. Personal information privacy and emerging technologies. Information Systems Journal.2013; 23:401–417.

